# Benign Breast Disease among Patients Visiting the Breast and Endocrine Clinic of a Tertiary Care Centre

**DOI:** 10.31729/jnma.8426

**Published:** 2024-02-29

**Authors:** Sishir Poudel, Sushan Pokharel, Sarada Khadka

**Affiliations:** 1 B.P. Koirala Institute of Health Sciences, Dharan, Sunsari, Nepal; 2 Department of Surgery, B.P. Koirala Institute of Health Sciences, Dharan, Sunsari, Nepal

**Keywords:** *benign*, *breast diseases*, *mastalgia*, *prevalence*

## Abstract

**Introduction:**

Breast diseases encompass a wide range of conditions, including benign and malignant disorders. Given the significant burden of breast-related health issues in the community, there is a critical need to understand the prevalence. This study aimed to find the prevalence of benign breast diseases among patients visiting the breast and endocrine clinic of a tertiary care centre.

**Methods:**

A descriptive cross-sectional study was conducted among patients presenting to the breast and endocrine clinic from 1 January 2022 to 1 January 2023 after obtaining ethical approval from the Institutional Review Committee. A convenience sampling method was used. The point estimate was calculated at a 95% Confidence Interval.

**Results:**

Among 979 patients, the prevalence of benign disease was 937 (95.71%) (94.44-96.98, 95% Confidence Interval). Mastalgia was the most frequent diagnosis 416 (44.40%), followed by fibroadenoma 137 (14.62%), benign lumps 84 (8.96%), and mastitis 64 (6.83%) and the most common symptoms reported by benign cases were pain in the breast 692 (73.85%) and breast lump 483 (51.55%).

**Conclusions:**

The prevalence of benign breast diseases was found to be similar to other studies done in similar settings.

## INTRODUCTION

The breast is a dynamic gland that experiences physiological changes throughout a female's reproductive years.^1^ Benign breast conditions include various lesions, such as developmental, inflammatory, and neoplastic.^2^ Hormones and growth factors influence both the epithelial and stromal components of the breast, resulting in Aberration in Normal Development and Involution (ANDI), which constitutes the majority of benign breast diseases.^3^

Global evidence supports a higher prevalence of benign breast diseases compared to breast malignancies. In Nepal, a study found that 61.7% were benign breast conditions, and 15.3% were malignancies.^3^ The estimation of the prevalence of benign breast disease in this setting is important in informing targeted healthcare interventions and improving diagnostic and management strategies for enhanced patient outcomes.

This study aimed to find the prevalence of benign breast diseases among patients presenting to the breast and endocrine clinic of a tertiary care centre.

## METHODS

This descriptive cross-sectional study was conducted among patients presenting to the breast and endocrine clinic of a B.P. Koirala Institute of Health Sciences, Dharan, Sunsari, Nepal. Ethical approval was obtained from the Institutional Review Board, BPKIHS (Reference number: 708/079/080-IRC). Data from 1 January 2022 to 1 January 2023 was collected between 1 July 2022 and 30 June 2023 from registration forms. Patients above 18 years who presented in the Surgical OPD with breast-related complaints were included in the study. Patients having missing or incomplete data were excluded from the study. A convenience sampling method was used. The sample size was calculated by using the following formula:


$$\begin{array}{ll} \text{n} & ={\text{Z}}^{2}\times \frac{\text{p}\times \text{q}}{{\text{e}}^{\text{2}}}\\ & =1.96^{2}\times \frac{0.578\times 0.422}{0.04^{2}}\\ & =586 \end{array}$$

Where,

n = minimum required sample size
Z = 1.96 at 95 % Confidence Interval (CI)
p = prevalence taken from a previous study, 57.8%^6^
q = 1-p
e = margin of error, 4%

The minimum required sample size was 586. However, the final sample size taken was 979.

The data collection involved a validated registration form extracted into a proforma with sections on sociodemographic profiles and contextual information related to presenting symptoms and family history. The study relied on thorough clinical history and physical examinations for initial clinical provisional diagnoses. Subsequently, essential investigations, including mammography, breast ultrasound and fine-needle aspiration cytology (FNAC), were conducted to validate the clinical diagnoses. Surgical intervention was reserved for patients requiring it, with excised specimens sent for histopathological examination.

Data were entered in Microsoft Excel 2019 and analysed using IBM SPSS Statistics version 11.5. The point estimate was calculated at a 95% CI.

## RESULTS

Among 979 patients, the prevalence of benign disease was 937 (95.71%) (94.44-96.98, 95% Confidence Interval). The mean age of the patients was 34.4±11.79 years. A total of 33 (3.52%) reported a family history of breast/ovarian malignancy. Mastalgia was the most prevalent diagnosis, with 56 (5.98%) diagnosed with cyclical mastalgia and 360 (38.42%) with non-cyclical mastalgia. Other common diagnoses included fibroadenoma in 137 (14.62%), benign lumps in 84 (8.96%), and mastitis in 64 (6.83%) (Table 1).

**Table 1:** Diagnosis of benign breast diseases (n = 937).

Diagnosis	n (%)
Mastalgia	416 (44.40)
Fibroadenoma	137 (14.62)
Benign Lump	84 (8.96)
Mastitis	64 (6.83)
Breast Abscess	46 (4.91)
Others	190 (20.28)

Pain was the most commonly reported symptom reported by 692 (73.85%) patients(Table 2).

**Table 2:** Symptoms of presentation among patients with benign cases (n= 937).

Complaints	n (%)
Pain	692 (73.85)
Lump	483 (51.55)
Nipple discharge	118 (12.59)
Nipple retraction	47 (5.02)
Sinus	2 (0.21)

## DISCUSSION

Among 979 female patients, the prevalence of benign breast disease was seen in 937 (95.71%). The prevalence of a past study conducted in a similar setting showed similar prevalence of 85.2% and 87.27%,^1,2^ while a study in similar settings in Nigeria showed a lower prevalence of 57.8% compared to our study.^4^

The study also found that the mean age of female patients was 35.29±12.77 years, with a mean of 34.4±11.79 years for benign disease. Benign breast changes are more common in women of childbearing age, peaking between the ages of 30 and 50.^5^ These findings are consistent with another survey study which showed a mean age for benign disease of 27±9.7 years.^4^

Regarding the presenting symptoms, 692 (73.85%) benign cases had pain as the presenting symptom. Furthermore, 483 (51.55%) benign cases found the lump as the presenting symptom and only 47 (5.02%) benign cases presented with retraction of the nipple. These findings were consistent with a study done in tertiary care hospitals in Nigeria,^4^ while the majority of other studies reported lump as the most common presenting symptom. Among the benign diseases, the most common diagnosis was mastalgia, followed by fibroadenoma which was inconsistent with other studies.^1,2,4,6^ Nevertheless, a study conducted in Nepal reported mastalgia as the most common diagnosis.^3^

The limitations of this study encompass potential selection bias in patient recruitment, reliance on self-reported data, and the single-centre nature of the study, which may affect the generalizability of the findings to broader populations and healthcare settings. Additionally, the study's retrospective design, lack of long-term follow-up data, and the absence of diversity in patient demographics are noteworthy limitations. Multicenter studies with larger sample sizes would be more suitable to generalize to our country's population and provide an accurate picture of benign breast diseases.

## CONCLUSIONS

The prevalence of benign breast diseases was found to be similar to other studies done in similar settings. Emphasis on community-based education programs targeting women within the reproductive age group, along with increased accessibility to pain management resources and early detection tools, could significantly contribute to improving overall breast health outcomes.
